# Differences in Production, Composition, and Antioxidant Activities of Exopolymeric Substances (EPS) Obtained from Cultures of Endophytic *Fusarium culmorum* Strains with Different Effects on Cereals

**DOI:** 10.3390/molecules25030616

**Published:** 2020-01-30

**Authors:** Jolanta Jaroszuk-Ściseł, Artur Nowak, Iwona Komaniecka, Adam Choma, Anna Jarosz-Wilkołazka, Monika Osińska-Jaroszuk, Renata Tyśkiewicz, Adrian Wiater, Jerzy Rogalski

**Affiliations:** 1 Department of Industrial and Environmental Microbiology, Maria Curie-Sklodowska University, Akademicka St. 19, 20-033 Lublin, Poland; renata.tyskiewicz@poczta.umcs.lublin.pl (R.T.); adrianw2@poczta.umcs.lublin.pl (A.W.); 2 Department of Genetic and Microbiology, Maria Curie-Sklodowska University, Akademicka St. 19, 20-033 Lublin, Poland; iwona.komaniecka@poczta.umcs.lublin.pl (I.K.); adam.choma@poczta.umcs.lublin.pl (A.C.); 3 Department of Biochemistry and Biotechnology, Maria Curie-Sklodowska University, Akademicka St. 19, 20-033 Lublin, Poland; anna.wilkolazka@poczta.umcs.lublin.pl (A.J.-W.); monika.osinska-jaroszuk@poczta.umcs.lublin.pl (M.O.-J.); rogal@poczta.umcs.lublin.pl (J.R.); 4 Military Institute of Hygiene and Epidemiology, Lubelska St. 2, 24-100 Puławy, Poland

**Keywords:** antioxidant activity, exopolymeric substances, *Fusarium culmorum*, sugar monomers, culture optimization, FTIR analyses

## Abstract

Exopolymeric substances (EPS) can determine plant-microorganism interactions and have great potential as bioactive compounds. The different amounts of EPS obtained from cultures of three endophytic *Fusarium culmorum* strains with different aggressiveness—growth promoting (PGPF), deleterious (DRMO), and pathogenic towards cereal plants—depended on growth conditions. The EPS concentrations (under optimized culture conditions) were the lowest (0.2 g/L) in the PGPF, about three times higher in the DRMO, and five times higher in the pathogen culture. The EPS of these strains differed in the content of proteins, phenolic components, total sugars, glycosidic linkages, and sugar composition (glucose, mannose, galactose, and smaller quantities of arabinose, galactosamine, and glucosamine). The pathogen EPS exhibited the highest total sugar and mannose concentration. FTIR analysis confirmed the β configuration of the sugars. The EPS differed in the number and weight of polysaccharidic subfractions. The EPS of PGPF and DRMO had two subfractions and the pathogen EPS exhibited a subfraction with the lowest weight (5 kDa). The three EPS preparations (ethanol-precipitated EP, crude C, and proteolysed P) had antioxidant activity (particularly high for the EP-EPS soluble in high concentrations). The EP-EPS of the PGPF strain had the highest antioxidant activity, most likely associated with the highest content of phenolic compounds in this EPS.

## 1. Introduction

Microbial polymeric substances (PS) have been studied for decades due to their interesting properties and novel functionality, which is not found in polymers produced by other organisms like algae or plants [1,2,3,4,5,6]. Polymers of microbial origin are commonly found in various natural environments (soils, waters, and air) [2,7,8]. These polymers, which are usually the main constituent of biofilms, enable microorganisms to colonize, adapt, and thrive in new ecological niches and under extreme physical, chemical, and biological (antagonistic micro- and macroorganisms and defense responses of plant or animal hosts) conditions [9,10,11]. While information on lighting conditions appears in studies on the production of EPS in algal cultures [12,13] and in few reports on bacteria of the genus *Rhizobium* [14], this problem is almost completely ignored in publications on the production of EPS in fungal cultures. Smirnov et al. [15] found that irradiation of *Cordoceps sinensis* inoculum slightly stimulated biomass biosynthesis, but significantly reduced exopolysaccharide production. Microbial PS are the key factors protecting cells against biotic and abiotic stress: drying, phagocytosis, and the action of antibiotics or toxic compounds [16]. EPS synthesis in fungal cultures is found in all growth phases [17,18]. It is generally accepted that EPS is produced in the case of a decrease in biomass [16] and under stress conditions [1,8,12], that do not promote biomass production but support sporulation, however, there are no publications directly related to the relationship between the production of EPS and the production of fungal spores. By their involvement in various interactions between organisms, also between microorganisms and plants, these compounds can determine the course of these interactions and the virulence of the microorganism producing them in relation to the host [19]. EPS can build the cell wall, constitute a backup substance, or be actively secreted into the external environment in the form of a capsule, slime or a biofilm. There are exo-, wall, endo-, and capsular PS. Exopolymers (EPS) are synthesized intracellularly during different growth phases and then secreted into the culture medium or are formed by lysis of cell and wall polymers [7,20]. EPS are synthesized by many groups of microorganisms such as bacteria, microalgae, diatoms, and fungi belonging to Asco-, Zygo-, and Basidiomycota [18,21]; however, the valuable biological properties of fungal EPS were recognized only 20 years ago [1]. Special attention in the last two decades has been focused on EPS synthesized by Ascomycota which inhabit many ecological niches and included saprotrophs colonising the rhizosphere, pathogens causing plant diseases, and non-pathogenic endophytes and symbionts of plants [22]. The synthesis of EPS is associated with secondary metabolism, and their structure and physicochemical properties depend on the type of strain, culture conditions, and composition of the substrate [23]. EPS have a large surface area and great diversity of structure, functional groups (carboxyl, phosphate, sulphate, amino, phenyl, hydroxyl) [24], sequences of monomeric units, glycosidic linkages, and different types of branching [21]. EPS are not homogeneous in their chemical composition and contain polysaccharides, i.e., usually the dominant component, and other equally important natural polymers that can play a key role, mainly proteins, phenolics, but also nucleic acids and lipids [1,25]. The main EPS component, polysaccharides, are a group of carbohydrates with a structure composed of at least ten monosaccharides connected with each other by α-glycosidic bonds (giving elasticity to the EPS chain) and β-glycosidic bonds (providing rigidity). They can be homopolymers composed of the same monosaccharides and heteropolymers consisting of two or more monomers [26]. Polysaccharides produced by microorganisms are divided into groups in various ways. Due to their location in the cell, they are divided into: (I) cytosolic, which are a source of carbon and energy, (II) cell wall components, and (III) external polysaccharides secreted into the environment [11]. Exopolysaccharides synthesized by fungi and bacteria represent a group of long chain, high molecular, linear and branched biopolymers [27]. The sugar subunits of polysaccharide components of microbial EPS are very diverse and often contain glucose, mannose, xylose, galactose, arabinose, rhamnose, fucose, and glucuronic acids as units of the main chains or branches [21,28,29]. 

Exopolymers have been widely used in the pharmaceutical, cosmetics, and food industries as stabilizers, thickeners, and gelling agents (e.g., curdlan, scleroglucan, pullulan) [30]. Fungal EPS have antioxidant, anti-cholesterol, and anti-cancer properties [21]. Due to the antioxidant properties and the ability to stimulate nerve cell growth, EPS has been used in medicine as potential auxiliary substances in nervous and autoimmune diseases [27]. As antioxidants, i.e., substances that can delay or inhibit oxidation, EPS can function in many ways, e.g., prevention of the formation of radicals, free radical scavenging, and formation of hydrogen peroxide and other peroxides and antioxidants [31]. Although over 400 different EPS have been structurally described, only a handful of these have been commercialized [32]. The biggest problem in the widespread use of EPS is usually their poor solubility in water or other polar solvents. The biological properties of EPS depend on both the biochemical composition and solubility of its individual components in various solvents [16,26]. EPS obtained from post-culture liquids of microorganisms show variable re-solubility in various solvents. In a sense, it is surprising that EPS present in dissolved culture liquids after being precipitated with alcohol are not re-soluble in water or even an ionic solvent such as DMSO [16]. Despite many advantages, many other potential applications and industrial use of EPS, the EPS synthesis process is still not fully understood [6,7]. Microbial culture techniques and methods for EPS production as well as their accurate quantitative and qualitative characteristics are constantly being improved [3,33,34,35,36]. New species of microorganisms capable of producing EPS with promising properties are being sought particularly among plant endophytic microorganisms [6,22,37,38,39,40,41]. Endophytic microorganisms, especially endophytic fungi, play an important role in plant ecosystems and have become the most promising source of antioxidant compounds [37,42]. Antioxidant properties of exo-metabolites produced by endophytic *Fusarium oxysporum* isolated from *Octoba gracilipes* leaves [43] and exopolysaccharides of *Fusarium equiseti* strain isolated from mangrove was detected [44].The involvement of bacterial EPS in symbiosis and plant pathogenesis has repeatedly been proven [45]. It is postulated, that microbial EPS plays an important role in plant protection against pathogens. Recently, interest in Ascomycota fungus exopolymers has been growing, especially due to their involvement in interactions with plants, *e.g.* fungi belonging to the genus *Fusarium* [18,46]. The ability to produce EPS has so far been described in *Trichoderma pseudokoningii* [47] and a few *Penicillium* species [21], *P. vermiculatum* [48], *P. citrinum* [49], and *P. paraphergal* [50]. *Fusarium moniliforme* (*Giberella fujikuroi*) EPS was obtained and characterized as early as in 1961 [51], but reports of the EPS of other *Fusarium* species have appeared in the last ten years: *F. coccophilum* strain BCC2415 [16], *F. solani* strain SD5 [52,53], and *F. oxysporum* strains Dzf17 [54] and Y24-2 [55]. EPS obtained from cultivation of *F. oxysporum* strain Dzf17 had elicitor properties against *Dioscorea zingiberensis* cells [54]. *Fusarium* represents a genus comprising more than 70 cosmopolitan species capable of producing a wide array of active metabolites [41,56], and their secondary metabolism has made the *Fusarium* species one of most important groups of fungi [57]. Most species from this genus are endophytes colonizing a large variety of plants species and exerting various effects on plants [58,59,60,61]. In addition to pathogenic strains that cause different types of fusariosis and produce such toxins as trichothecenes, fumonisins, zearalenones, and fusarin as well as cell wall degrading enzymes (CWDE) [62,63,64], there are also plant growth stimulating strains producing phytohormones [65]. The present research is a continuation and extension of previous experiments on obtaining and characterizing cell wall polysaccharides extracted from cultures of endophytic *Fusarium culmorum* strains differently affecting the growth of cereal plants [63,64]. According to the type of their interaction with plants, *F. culmorum* strains can be divided into plant growth promoting fungi (PGPF), harmful deleterious rhizosphere microorganisms (DRMO), and pathogenic strains. The interaction of these strains with cereal plants has been well studied and described [58,59]. It has also been shown that polysaccharides derived from the cell wall of *F. culmorum* strains [63,64] have the ability to induce cereal resistance against fusariosis, i.e., they have the same elicitor properties [58] as the mycoparasitic *Trichoderma* DEMTkZ3A0 strain [60]. 

It has been shown that pathogenic and non-pathogenic strains with different types of effects on plants are present within such species as *F. oxysporum* and *F. culmorum* [58,59,66,67,68]. It is postulated that the type of interaction depends on the degree of colonization of roots, stems, and border cells and the synthesis of toxins, CWDE, phytohormones and, indirectly, through induction of plant resistance. It can therefore be assumed that EPS produced by *F. culmorum* strains may have an impact on the type of interactions with cereal plants directly or indirectly showing elicitor properties like fragments of cell walls of these fungi. There are no studies showing differences in the properties of polymers produced by endophytic fungal strains of the same species exerting different effects on plants.

The aim of the study was to compare production of exopolymers by three *Fusarium culmorum* strains exerting different effects on plants —a DRMO, a PGPF and a pathogenic one—and to determine the differences between the EPS obtained from culture of these strains, with particular emphasis on their biochemical properties, composition, and dissolution and antioxidant ability. 

## 2. Results and Discussion

The ability of *F. culmorum* strains to synthesize EPS and the efficiency of the synthesis in different growing conditions were tested for the three individual strains: DEMFc2, DEMFc5, and DEMFc37. 

### 2.1. EPS Isolation and Fractionation 

**Figure 1:**
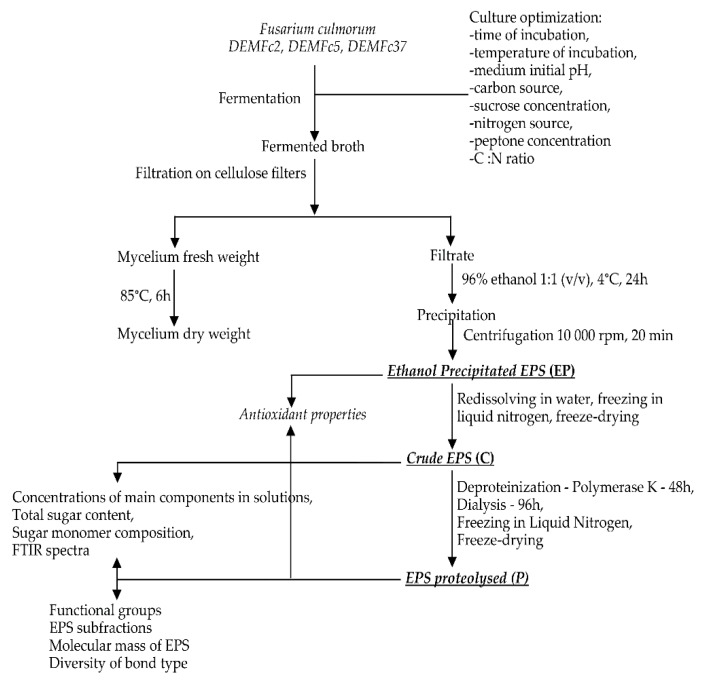
Scheme for the procedure of isolation and fractionation of EPS from liquid cultures of the three *Fusarium culmorum* strains (DEMFc2, DEMFc5 and DEMFc37) in three forms: Ethanol Precipitated (EP)**,** Crude (C)***,*** and Proteolysed EPS (P) and the protocol for studies of EPS properties.

**Figure 2:**
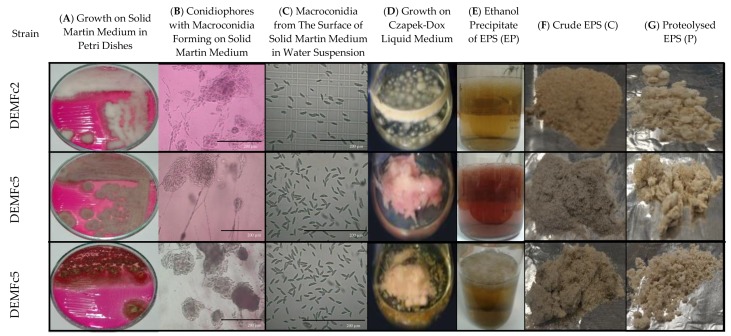
Macrographs (**A**, **D**, **F**, **G**) and LM micrographs (**B**, **C**) documenting the steps of the procedure of obtaining three forms of EPS from three *Fusarium culmorum* strain cultures.

Exopolymers (EPS) were obtained from liquid cultures on Czapek-Dox medium after inoculation with macroconidia of *F. culmorum* strains (Figure 1, Figure 2). First, optimal conditions for obtaining EPS were selected. Then, biochemical properties were determined for EPS preparations obtained from cultures kept in optimal conditions. Three forms of EPS were tested: a preparation obtained directly after alcoholic precipitation-ethanol precipitated EPS (EP), a preparation subjected to freezing in liquid nitrogen and lyophilisation referred to as crude EPS (C), and finally a preparation subjected to the process of proteolysis, proteolysed EPS (P) (Figure 1, Figure 2). 

**Figure 3:**
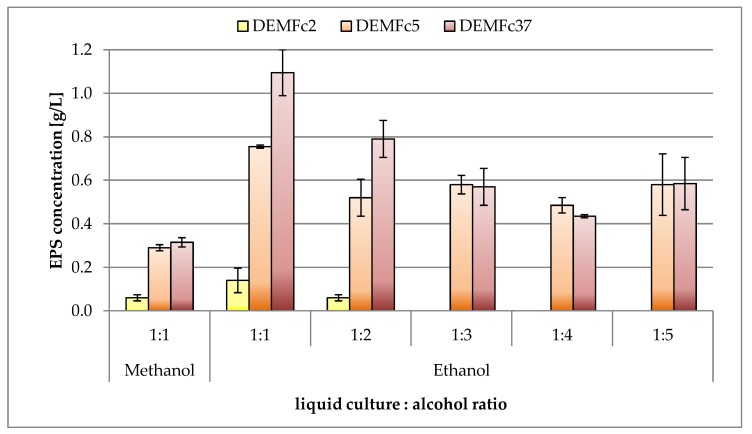
EPS precipitation procedure using different alcohols (methanol, ethanol) and different liquid culture:alcohol ratio.

The solubility of the EPS obtained in water, alkaline, and acidic solutions was assessed, and fractionation of the obtained EPS was carried out for two forms of EPS: crude (C) and proteolysed (P) (Figure 1, Figure 2). The content of protein, phenolic compounds, and sugars as well as the composition of sugar monomers were determined in two forms of EPS: C and P. These two forms of EPS were subjected to FTIR analysis. The EPS antioxidant properties were checked for these two forms and also for the third form – ethanol precipitated (EP). The EPS precipitation procedure with alcohol and 1:1 liquid culture:alcohol ratio was selected in preliminary studies (Figure 3).

EPS alcohol precipitation methods usually consist in the use of 95% ethanol or absolute ethanol in an ethanol volume usually exceeding three times the volume of post-culture fluid, and in many cases even four or five times [18]. At EPS precipitation, we have shown that the most effective is the use of an equivalent (1: 1) ratio of culture supernatant to alcohol (ethanol) (Figure 3). The method of obtaining EPS based on its precipitation from post-culture liquids using ethanol has been refined [18,47], and the EPS pre-treatment method involved deproteination and dialysis. 

### 2.2. Optimization of the EPS Production

**Figure 4:**
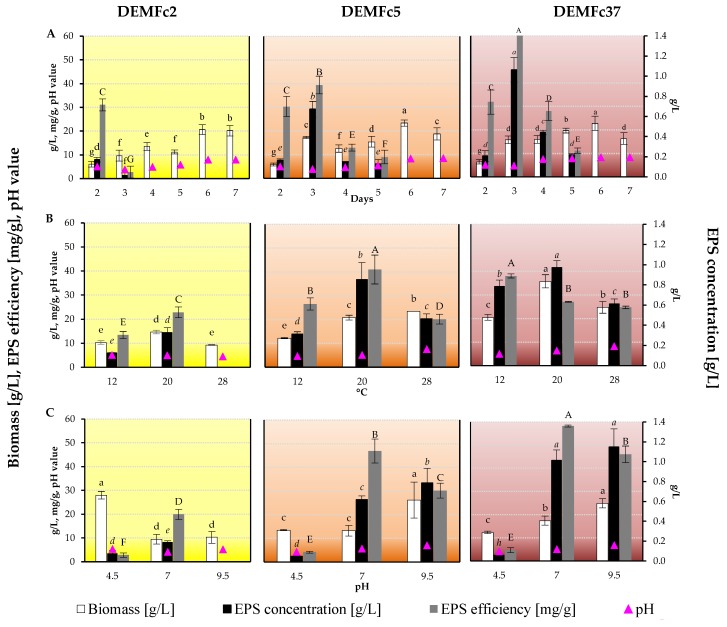
Fungal dry biomass [g/L], EPS yield: EPS concentration [g/L], EPS concentration converted into fungal biomass dry weight [mg/g], and final pH of the post-culture liquid at different condition of: (**A**) time of incubation, (**B**) temperature of incubation, (**C**) medium initial pH value of *F. culmorum* cultures. Statistical data analysis: one-way ANOVA with post hoc Tukey’s HSD test, *p* < 0.05. Mean values labeled with the same letter within the same series are not significantly different (*p* > 0.05). Standard deviations are shown as deviation bars (*n* = 9).

The investigations indicate that the ability to synthesize EPS by three tested *F. culmorum* strains and the properties of EPS are strain-specific features. The EPS yield was expressed as the concentration per liter (g/L) of liquid cultures on Czapek-Dox medium with sucrose (S) and peptone (P) and converted into EPS production effectiveness — the concentration calculated per g of fungal biomass (Figure 4). The highest yield was obtained for EPS in the DEMFc37 strains and the lowest value was noted for EPS in the DEMFc2 culture. During the 2–7 days of culturing, the highest EPS yield values were obtained in 2-day cultures of the DEMFc2 and DEMFc5 strains and in 3-day cultures of the DEMFc37 strain. On the optimal EPS production days, the highest EPS yield was obtained from cultures kept at 20 °C. The highest EPS yield values were obtained in cultures of the DEMFc5 and DEMFc37 strains on medium with an initial pH value of 7 and 9.5, but the efficiency was higher at pH 7. However, in the culture of the DEMFc2 strain on a medium with an initial pH of 9.5, no EPS was recorded in the post-culture liquid medium. In optimal conditions for EPS production, the highest biomass values were usually not recorded; except for the 3-day DEMFc5 culture, the highest value was noted in the 6-day cultures. The pH value dropped below the initial pH value of the medium on the third day of culture, after which it gradually increased on the following days to reach a maximum on the seventh day.

The amounts of EPS obtained from liquid cultures of three endophytic *Fusarium culmorum* strains (DEMFc2, DEMFc5, DEMFc37) with different aggressiveness towards cereal plants (growth promoting-PGPF, deleterious-DRMO, and pathogenic) varied and depended on many factors: the source and concentration of carbon and nitrogen, temperature, pH value of the medium, and the period of incubation. The highest biomass of *F. culmorum* mycelium was obtained in much older (6 and 7 days) cultures than those in which the highest EPS concentrations (2 and 3 days) were obtained. However, on the optimal day for EPS production, it was found that the optimum temperature for EPS production of 20 °C is also the most favourable for biomass production. Similarly, the pH value of 9.5 was optimal on the third (optimal) day of culture for obtaining both the largest biomass and the largest EPS yield of DEMFc5 and DEMFc37 strains. Often, increases in EPS have been obtained at different culture incubation times [18] in the case of a decrease in biomass [16] and under stress conditions [1,8,12]. The maximum concentration of EPS in the post-culture liquid of the tested strains was close to the maximum values known from the literature on various types of Ascomycota fungi [16,21]. 

The maximum EPS yield varied in a wide range (from 0.1 g/L to 10 g/L) in Basidiomycota cultures, and a particularly wide range of yield was observed in Ascomycota fungi cultures (from 0.1 g/L to 40 g/L for *Aureobasidium pullulans* [18,69]). In the cultures of fungi of the genus *Fusarium* spp. studied so far, the EPS yield differences were more than 10-fold from 0.12 for *F. oxysporum* strains to 2.83 for *F. coccophilum* [16,18,55,70]. An approximately 5-fold difference in the maximum yield was found between *F. oxysporum* strains [18,55,70,71,72]. The *F. solani* DO7 strain produced approx. 2.8 g EPS/L in optimal conditions [18]. This value in *Fusarium oxysporum* described in the literature was in the range of 0.12–0.59 g/L [54,55,72]. However, by appropriate modifications of the composition of the substrate, i.e., carbon and nitrogen sources and their concentrations in a *Fusarium solani* strain culture, Mahapatra and Banerjee [21] achieved a several times higher level of EPS synthesis (approx. 2.3 g/L).

**Figure 5:**
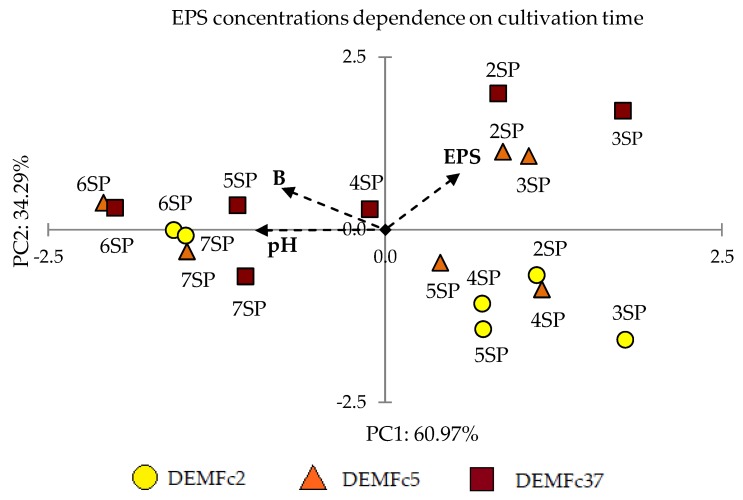
Principal component analysis (PCA) - dependence of EPS concentrations (EPS), fungal dry biomass (B), and pH values in post-culture liquid on the cultivation time in cultures of *F. culmorum* strains with sucrose (S) and peptone (P) as an C and N source, respectively.

The principal component analysis (PCA) applied for assessment of the concentration of EPS and fungal biomass and the pH values in the post-culture liquids indicates that high EPS yield values are not accompanied by high biomass concentration values or high pH values (Figure 5). 

The PCA analysis clearly demonstrates that the highest EPS concentration was obtained in the young (2- and 3-day) cultures of the *F. culmorum* Fc5 and Fc37 strains. The highest biomass was recorded in 6-day cultures of these strains and the value was only slightly lower in the Fc2 strain culture. The pH value was positively correlated with the culture age and biomass size. The PCA analysis based on the EPS and biomass concentrations and pH values in post-culture liquid showed a strong separation of the samples into two groups – group I: 2SP-5SP (except 4 and 5SP for Fc37) and group II: 6-7SP. The variables from the first group are positively correlated with axis 1. The first two components of the PCA analysis explained 95.26% of the total variance. The first component was negatively loaded mainly by pH and by biomass concentration. The first axis of the PCA diagram showed the gradient of pH correlated with the cultivation time and the fungal biomass. The left side of the diagram focused on the low pH and high biomass for samples from the long-term culture (6 and 7SP). The highest biomass of 6SP was correlated with the lowest pH values. The right side of the diagram showed a relationship between the high EPS concentration and the shorter age of the culture, i.e., the highest EPS yield was determined for 3-day cultures.

**Figure 6:**
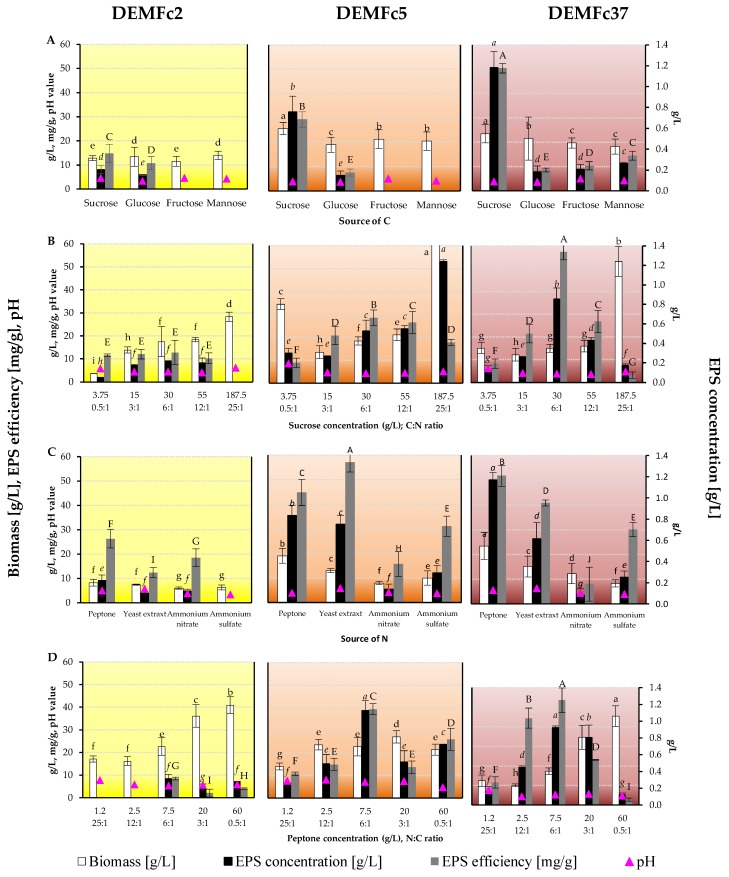
The EPS concentration in *F. culmorum* strain culture on: (**A**). carbon source, (**B**). sucrose concentration (g/L), C:N ratio, (**C**). nitrogen source, (**D**). peptone concentration (g/L), N:C ratio. One-way ANOVA with post hoc Tukey’s HSD test, *p* < 0.05. Mean values labeled with the same letter within the same series are not significantly different (*p* > 0.05). Standard deviations are shown as deviation bars (*n* = 9).

Appropriate selection of substrate components, both carbon and nitrogen sources, as well as micro- and macroelements can lead to intensification of EPS production in fungal cultures (Figure 6).

Usually attention is focused ono the carbon source, with glucose [72], sucrose [73] or maltose [74] as the most common substances, and the nitrogen source, for example organic substances such as peptone [72], yeast extract [50], or mineral salts, e.g., NaNO_3_ [48]. The presence of micro- and macroelements: MgSO_4_, KH_2_PO_4_, and FeSO_4_, in the medium is necessary for the growth of microorganisms, and a change in their composition and concentration may induce changes in the intensity of EPS production [48,50,72,73,74].

It should be taken into consideration that both the composition of the medium and the optimal culture conditions for EPS synthesis are not the same as the optimal ones for strain growth. The highest effectiveness of EPS synthesis may be recorded in stress conditions, as EPS can be an element of adaptation to these conditions.

Both EPS production efficiency and composition may be determined not only by the type of substrate constituting the C and N source but to a large extent by the C:N ratio. A significant change in the C:N ratio may cause disorders in Ascomycota fungi growth and development, which may result in a defence reaction. Literature data indicate a significant impact of the C:N ratio on the amount of EPS synthesized [75,76]. However, the data on the optimal C:N ratio value for EPS synthesis and the correlation between the EPS concentration and the C:N ratio value are often contradictory. Most likely, the optimal C:N ratio depends not only on the species and even the strain, but also on the source of C and N itself.

There are data indicating that values of the C:N ratio lower than 1 are optimal for EPS synthesis: the value of the 0.5 in the culture of *Chrysobacterium daegenes* [77] or 0.8 in the mycorrhizal fungus *Tricholoma matsutake* belonging to Basidiomycota [78]. 

In other studies, the C:N ratio of 5 was optimal for EPS synthesis, as observed in the culture of *Ganoderma lucidum* [79] or exceeded this value, as in the culture of *Lactobacillus fermentum* in a medium with the C:N ratio of 6.3 [80]. On the other hand, a C:N ratio of 20 was reported to be optimal for EPS production in active sludge [81]. 

At a C:N ratio of about 5, the content of proteins in the EPS from activated sludge was relatively high and began to decrease as the value of the C:N ratio increased, which was accompanied by an increase in the content of polysaccharides in the activated sludge EPS [82].

The optimal incubation period of cultures for EPS synthesis was determined and the effect of various carbon and nitrogen sources on the amount of EPS synthesized was assessed, selecting Czapek-Dox medium (CD) with 3% (30 g/L) sucrose (Figure 6A) and 0.75% peptone (Figure 6C), in which the C:N and N:C ratio was 6:1 (Figure 6B, Figure 6D), as a standard for all strains tested.

The composition of the medium can be modified by lowering and increasing the sucrose concentration (compared to the standard 3%) at a constant peptone concentration (experimentally selected 0.75%) and lowering and increasing the peptone concentration at a constant 3% sucrose concentration, the use of the different sucrose and peptone concentrations yielded a C:N ratio in the range from 0.5 to 25 in the media.

In the Czapek-Dox medium with sucrose as a C source, the EPS concentration was about 5-, 3-, and 1.5-fold higher for the Fc37, Fc5, and Fc2 strains, respectively than in the glucose medium (Figure 6A). No EPS production was recorded in the Fc5 and Fc2 strain culture on a medium with fructose or mannose. Sucrose was the optimal sugar for the biomass increase noted in the Fc5 and Fc37 strains, and the biomass of the Fc2 strain obtained in sucrose-supplemented culture differed slightly or statistically insignificantly from the biomass obtained in media with the other sugars (Figure 6B). The sucrose concentration in the medium proved to be the best for the efficiency of EPS production by the Fc37 and Fc2 strains and for the EPS yield in the post-culture fluid. In the case of the EPS strain Fc37, the effectiveness in the medium with 3% sucrose was over 10 times higher and the yield was about 5 times higher than at the other sucrose concentrations. In the case of the Fc5 strain culture, the highest EPS yield was obtained at a sucrose concentration of 187.5, i.e., at a C:N ratio of 25. In these conditions, however, no EPS production by the Fc2 strain was recorded. The highest amount of biomass of the three strains was noted at the highest sucrose concentration. In the presence of peptone as an N source in the media, the cultures of the three tested strains had the highest EPS yield, and the efficiency of EPS formation was only slightly higher in the Fc5 cultures with yeast extract as an N source (Figure 6C). The highest EPS yield and efficiency of production thereof in the cultures of each of the three strains were recorded when the peptone concentration was 7.5 and the N:C ratio was 6:1 (Figure 6D). EPS was not obtained in Fc2 cultures containing peptone at a concentration of 1.2 and 2.5. A thorough review of the state of knowledge on the optimization of EPS production, which we previously presented in a review publication [18], indicates that most often the optimal C source for EPS production in Asco- and Basidiomycota fungi was glucose, usually used at a concentration of 20 g/L, but in many studies the optimal concentration was 50 g/L. Less often, optimal conditions for the production of EPS were created by the introduction of such C sources as maltose, mannitol, sorbitol, and PDB (potato dextrose broth). Sucrose was usually used in high (40-50 g/L) concentrations, but 10 g/L proved optimal for Ascomycota strain *Hypocreales* sp. NCHU01 [18,83]. In contrast, Ascomycota yeast *Cryptococcus laurentii* [18,84] produced EPS most efficiently at a concentration of 35 g/L, i.e., close to the optimum determined for EPS production in *F. culmorum* strain cultures (30 g/L). In most fungal cultures, the best source of N was yeast extract, usually used at a concentration of about 3 g/L, but also frequently combined with other N sources, e.g., ammonium sulphate or peptone [18].

In our research, we found that EPS production by *F. culmorum* strains is most effective at the species-specific concentration and the presence of appropriate amounts of C (sucrose - 3.0%) and N (peptone - 0.75%) and initial pH value (7.0). The clear difference between EPS production by *F. culmorum* strains and other filamentous or yeast Asco- or Basidiomycota strains concerns the optimal day of culture, which is day 2/3 for *F. culmorum*, but day 5–7 and even day 30 for most fungi, e.g., for another Ascomycota species - *Penicillium griseofulvum* [18,85].

Also, the initial pH value of 7.0 determined for *F. culmorum* cultures as optimal for EPS production was higher than in the case of most fungi [18]. 

**Figure 7:**
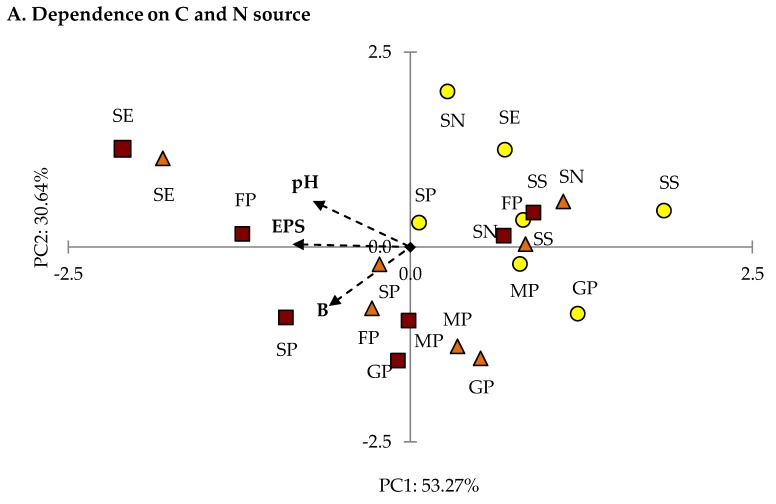

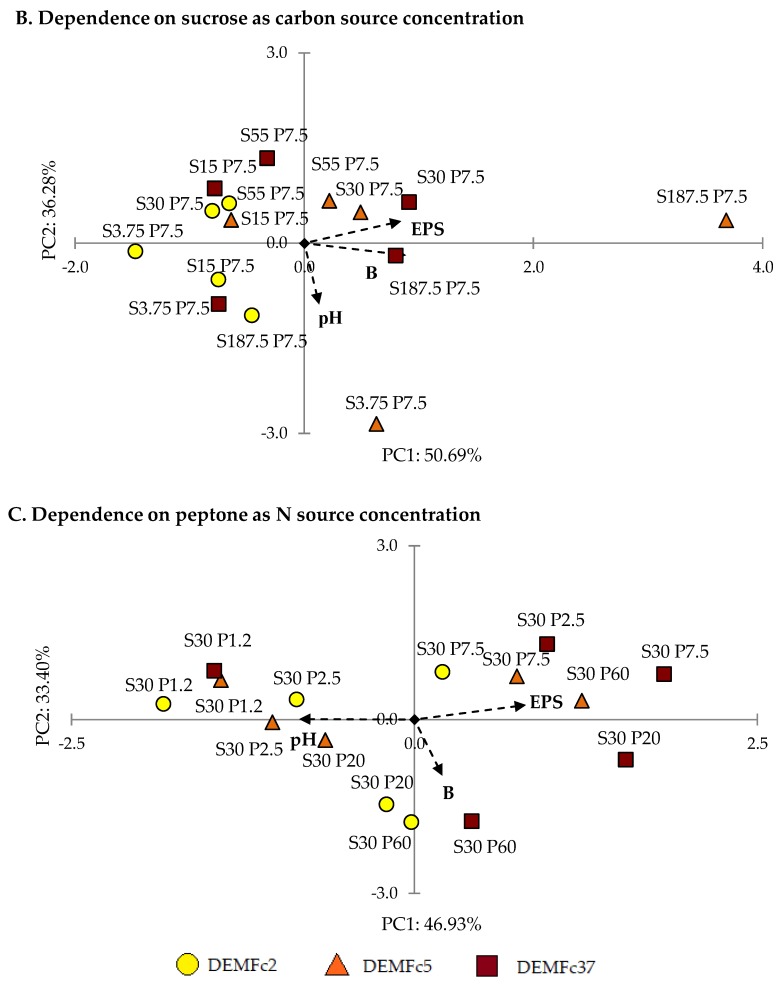
PCA-dependence of EPS concentrations (EPS), fungal dry biomass (B), and pH values in post-culture liquid on: (**A**) C and N source type, (**B**) sucrose concentration (g/L), (**C**) peptone concentration (g/L) in *F. culmorum* strains culture. Descriptions: S – sucrose, P – peptone, G – glucose, F – fructose, M – mannose, N – ammonium nitrate, S - ammonium sulphate; the numbers correspond to the concentration of C and N sources.

The results of the optimization of EPS production depending on the source C and N and the concentration of these sources subjected to PCA analysis showed that the values obtained for the Fc2 strain were positively correlated with axis 1 (Figure 7A).

The first two axes of PCA analysis explained 83.91% of data variability. EPS concertation, pH, and biomass concentration were negatively correlated with axis 1. The diversification between the distribution of the DEMFc37 and DEMFc samples was low. The variables with SN (sucrose and ammonium nitrate) were positively correlated with axis 2. Based on the EPS concertation, the lowest concentration was achieved for the Fc37 and Fc5 culture on SE (sucrose and yeast extract) and for Fc37 on FP (fructose and peptone).

The first two components of the PCA analysis explained 86.97% of the total variance (Figure 7B). All yellow samples (for the Fc2 strain) were negatively correlated with axis 1. Both concentrations — biomass and EPS were positively correlated with axis 1 and pH exhibited a negative correlation with axis 2. The highest EPS concentration was achieved for S187.5 P7.5.

The first two axes of the PCA analysis explained 80.33% of data variability (Figure 7C). EPS concentration was positively, and pH negatively correlated with axis 1. Biomass concentration was negatively correlated with axis 2. The Fc37 samples were mostly positively correlated with axis 1, whereas a negative correlation was found for the Fc2 samples. The Fc5 samples were evenly distributed in the plot. The highest EPS concentration for S30P7.5 was related to the pH values. The highest pH value was detected for S30 P1.2 with the lowest EPS concertation or absence of EPS in the post-culture liquid.

### 2.3. Composition of the EPS Solution in Three Solvents (Water, Sodium Base, Acid) and Total Sugar Content of EPS

**Figure 8:**
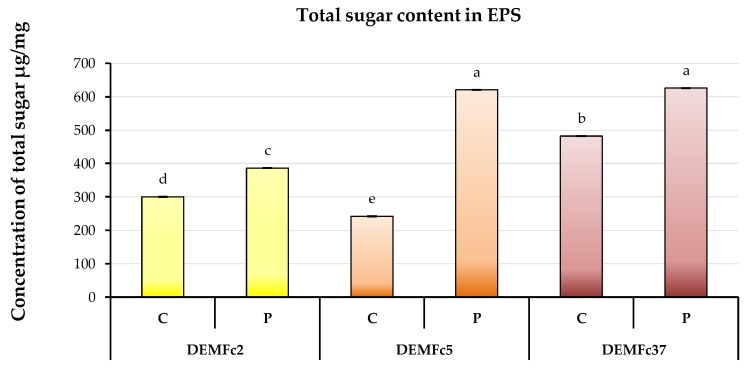
Total sugar in crude (**C**) and proteolysed (**P**) EPS obtained from cultures of three *F. culmorum* strains: DEMFc2, DEMFc5, and DEMFc37. ANOVA with post hoc Tukey’s HSD test, *p* < 0.05. Mean values labeled with the same letter are not significantly different (*p* > 0.05). Standard deviations are shown as deviation bars (*n* = 9).

The total sugar content in crude EPS reached a value close to 500 µg/mg dry mass in the case of EPS obtained from the Fc37 strain culture (Figure 8). It was twice lower in the crude EPS from strain Fc5 After proteolysis of the EPS preparations, an increase in the total sugar content in proteolysed EPS (P) was found, in comparison to crude (C). The content of sugars in EPS obtained from the cultivation of strains Fc5 and Fc37 was almost identical and amounted to over 600 µg/mg dry mass. It was by 1/3 lower in the EPS from Fc2. The crude EPSs (C-EPS) of *F. culmorum* were contained 30, 24, and 48% (*w/w*) of sugars and after proteolysis (P-EPS) percentage increased to 38, 62, and 62,5% (*w/w*) in the Fc2, Fc5, and Fc37 strains, respectively.

**Figure 9:**
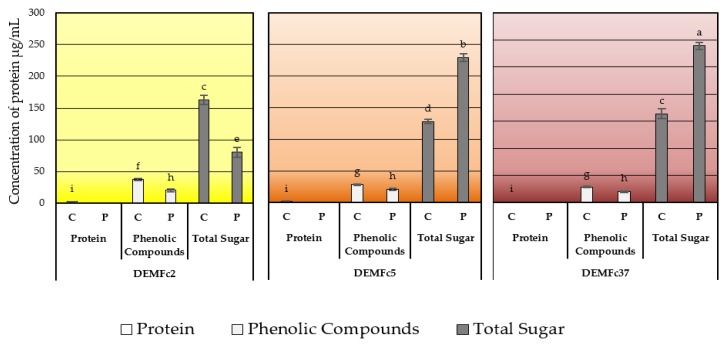
Concentration of protein, phenolic compounds and total sugar in water solution of crude (**C**) and proteolysed (**P**) EPS obtained from cultures of three *F. culmorum* strains: DEMFc2, DEMFc5, and DEMFc37. ANOVA with post hoc Tukey’s HSD test, *p* < 0.05. Mean values labeled with the same letter within the same series are not significantly different (*p* > 0.05). Standard deviations are shown as deviation bars (*n* = 9).

In the water solution the highest concentrations of sugars were detected, especially in the EPS (P) of the Fc5 and Fc37 strains (Figure 9). In the case of strain Fc2 after proteolysis, the content of sugars in the aqueous solution decreased almost two times compared to the content in the crude EPS. The content of sugars in the water fraction was up to 10 times (Fc37) higher than the content of phenolic compounds. The highest concentrations of phenolic compounds in water solution were detected in the crude EPS of strain Fc2. In contrast, the content of these compounds was the lowest in the EPS (P) preparation of the Fc2 strain. 

The solubility of EPS is a strain-specific trait and must be determined before carrying out analyses to determine the physical, chemical, and biological properties of these polysaccharides [16]. The EPS of *F. cocophillum* strain BCC2415 was only partially water-soluble and insoluble in DMSO. It should be emphasized that the exopolymer of this strain also had a different composition. It contained less than 20% of sugars and over 60% of protein, while the content of sugars in other studied fungal EPS ranged from 40% to about 90%, and the protein content was usually 1–10% [16]. 

**Figure 10:**
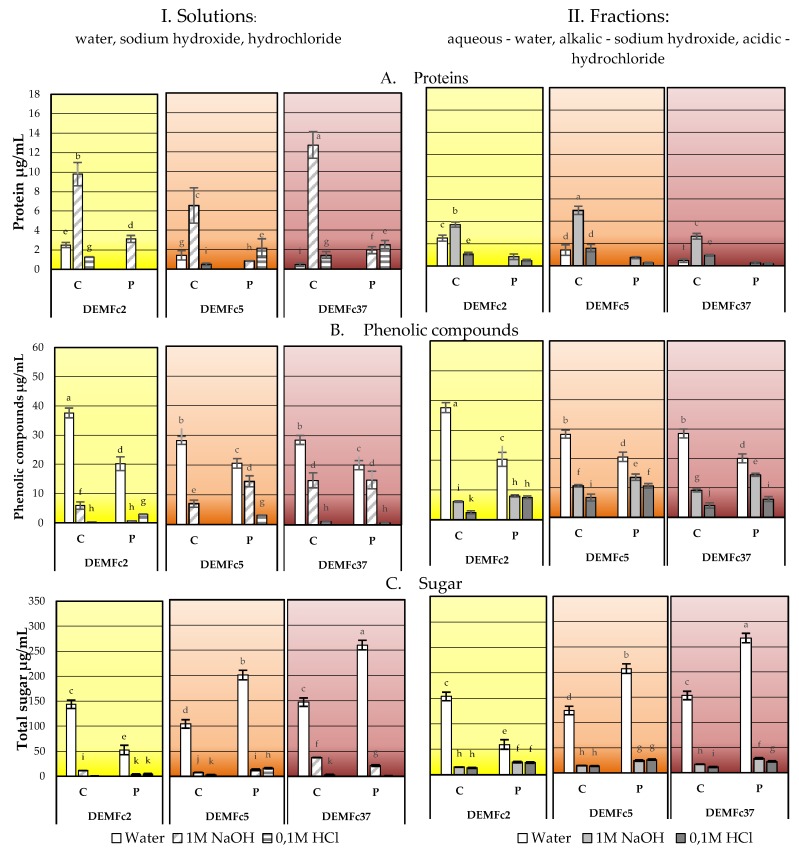
Concentration of protein (**A**), phenolic compounds (**B**) and total sugar (**C**) in (**I**). Solutions (water, sodium hydroxide, hydrochloride) obtained by dissolving EPS directly in each of the three solvents and (**II**). fractions (aqueous-water, alkalic-sodium hydroxide, acidic-hydrochloride), obtained in step fractionation of crude (C) and proteolysed (P) EPS obtained from cultures of three *F. culmorum* strains: DEMFc2, DEMFc5, and DEMFc37. ANOVA with post hoc Tukey’s HSD test, *p* < 0.05. Mean values labeled with the same letter within the same series are not significantly different (*p* > 0.05). Standard deviations are shown as deviation bars (*n* = 9).

**Figure 11:**
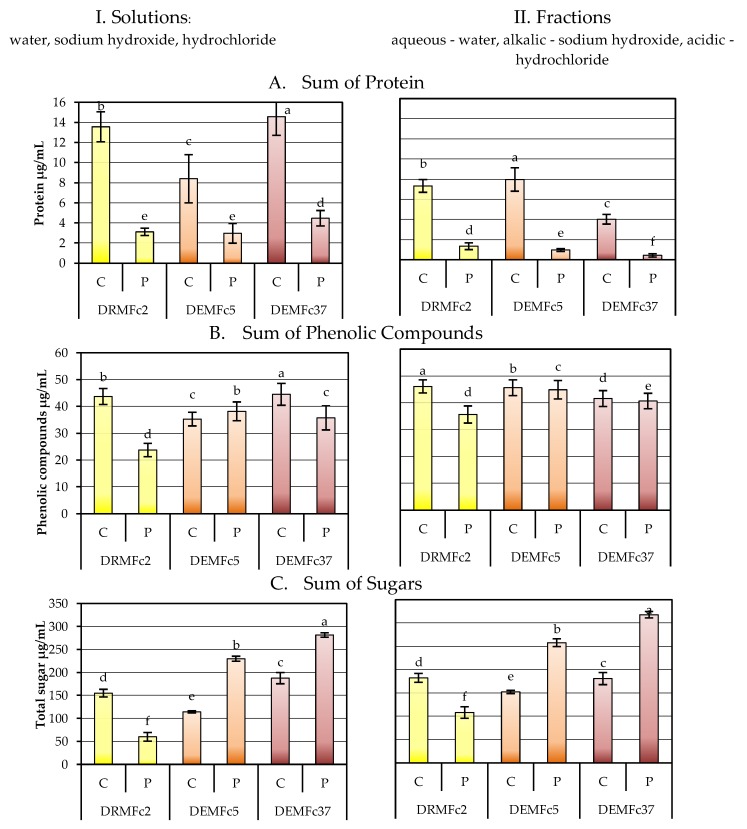
Sum of protein (**A**), phenolic compounds (**B**) and total sugar (**C**) in three I. Solutions (water, sodium hydroxide, hydrochloride) obtained by dissolving EPS directly in each of the three solvents and three II. fractions (aqueous-water, alkalic-sodium hydroxide, acidic-hydrochloride) obtained in step fractionation of crude (C) and proteolysed (P) EPS from cultures of three *F. culmorum* strains: DEMFc2, DEMFc5, and DEMFc37. ANOVA with post hoc Tukey’s HSD test, *p* < 0.05. Mean values labeled with the same letter within the same series are not significantly different (*p* > 0.05). Standard deviations are shown as deviation bars (*n* = 9).

The content of sugars, proteins and phenolic compounds in water, NaOH, and HCl solutions obtained by dissolving EPS directly in each of the three solvents (I) and by gradual (step) fractionation (II) was determined (Figure 10) and sum of concentration of these compounds dissolved in these three solvents directly (I) or by fractionation (II) was demonstrated in Figure 11. 

Crude and proteolysed EPS preparations were analysed (Figure 10, Figure 11). Protein was mainly present in the crude EPS alkaline solutions, with the highest level in the EPS of Fc37 and the lowest amounts in the Fc5 culture. Three- to six-fold lower protein concentration was detected in the acid fractions of proteolysed EPS (P) obtained from the Fc5 and Fc37 cultures and the Fc2 and Fc37 alkaline fraction than in the crude EPS alkaline fraction, and no protein was detected in the EPS (P) aqueous fraction. 

When crude and proteolysed EPS was dissolved directly (Figure 10.I) in three separate solvents, the protein was detected, as in the step fractionation (Figure 10.II), mainly in the alkaline solution, but content of the protein in EPS of the Fc2 and Fc37 strains decreased. The highest concentrations of phenolic compounds were found in the solution of crude EPS of strain Fc2. The same concentrations were detected in the crude EPS of Fc5 and Fc37. These compounds also dissolved in the alkaline and acid solution but to a much lower degree than in water.

Total sugars were detected in the crude and proteolysed EPS aqueous solutions at concentrations more than 10 times higher than in the alkaline and acidic solutions (Figure 10.I). In total, phenolic compounds (PCs) were present at similar concentrations in EPS of three strains, although their content was statistically the highest in the EPS of Fc2 water solution and aqueous fraction (Figure 10) and sum of PCs fractions in Fc2 strains EPS was also highest (Figure 11.II). Only in the case of the EPS from strain Fc2, the content of sugars in the proteolysed EPS decreased compared to the content in the crude EPS (Figure 10, Figure 11). 

Madla et al. [16] found that only seven out of 16 EPS produced by strains belonging to separate Asco- and Basidiomycota species were completely dissolved in water. EPS obtained from the culture of various *Botryosphaeria rhodina* strains was found to be soluble not only in water but also in 1M NaOH [73,86]. EPS produced by a strain belonging to the genus *Hypocreales* sp. dissolved in 1M NaOH at 60 °C [83], and EPS from the culture of *Morchella crassipes* was soluble in 0.2M NaCl [74].

#### Sugar Composition of the EPS

**Figure 12:**
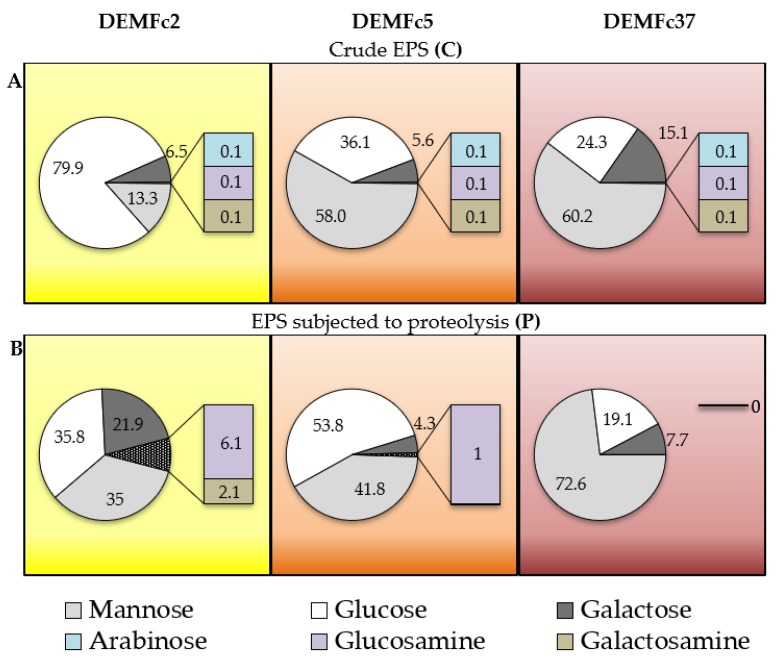
Percentage (%) content of sugar monomers (mannose, glucose, galactose, arabinose, glucosamine and galactosamine) in: (**A**) crude EPS (C) and (**B**) proteolysed (P) EPS obtained from cultures of three *F. culmorum* strains: DEMFc2, DEMFc5, and DEMFc37.

The content of sugar (Figure 12) in the crude EPS was different than in the proteolysed EPS. Especially, in the EPS-P, the number of detected sugars decreased. The estimated glucose contents in crude EPSs were 80, 36, and 24%. They changed significantly after proteolysis of EPS and reached 38, 54, and 19% for the EPS of the Fc2, Fc5, and Fc37 strains, respectively. In the P-EPS of the pathogenic strains, the mannose content increased to 73% (60% in the crude EPS). 

The EPS produced by *F. culmorum* consists mainly of glucose, mannose and galactose. The crude EPS of the Fc2 strain is dominated by glucose (79.9%), and mannose (13.3%) (Figure 12A). The last-mentioned sugar constitutes about 60% of the sugar fractions in the EPS (C) of the Fc5 and Fc37 strains. The highest galactose content was found in the crude EPS from Fc37. Approximately 0.1% of arabinose, glucosamine, and galactosamine were also present in the crude EPS of each of the tested strains. In the case of proteolysed EPS, the largest change in the monomer sugar composition was recorded in the Fc2 EPS (Figure 12B). Its EPS (P) exhibited an over two-fold, three-fold and almost four-fold increase in the content of glucose, mannose, and galactose, respectively. It appears that glucose or glucose oligomers present in the crude EPS from Fc2 strain may have been bound to the protein and was removed from EPS preparation during it digestion with proteinase. In the EPS (P) of strain Fc37, the dominant (72.6%) sugar was mannose, while glucose was the main sugar in the Fc5 EPS (P). Only three hexoses were detected in the EPS (P) of Fc37. 1% glucosamine was detected in the EPS (P) of Fc5, whereas the EPS (P) of Fc2 contained as much as 6% of glucosamine and 2% galactosamine. 

The polysaccharide parts of fungal EPS are heteropolymers and homopolymers usually composed only of glucose monomers [87]. Heteropolymers typically contain 2–3 sugar monomers, which are usually mannose and galactose besides glucose. In the EPS of Basidiomycota fungi, in addition to the three sugars mentioned above, xylose and rhamnose as well as glucuronic acid are common as well [87]. Galactomannans of filamentous fungi occurring in the form of fungal-type (FTGM) and O-mannose type are important in infection and defence mechanisms and, as fungal antigens, they can play a key role in the diagnosis of infection caused by e.g., *Aspergillus* or *Fusarium* spp. species [87]. By comparing the composition of EPS obtained in *F. solani* DO7 cultures, Zeng [6] found that the composition of monosaccharides and their molar ratio depend on the C:N ratio in the medium. The monosaccharide composition of *F. culmorum* EPS was studied in the present work for EPS obtained in cultures of three strains of *F. culmorum* on a medium with a C:N ratio of 6.

### 2.4. Characterization of EPSs

#### 2.4.1. FTIR Spectra 

**Figure 13:**
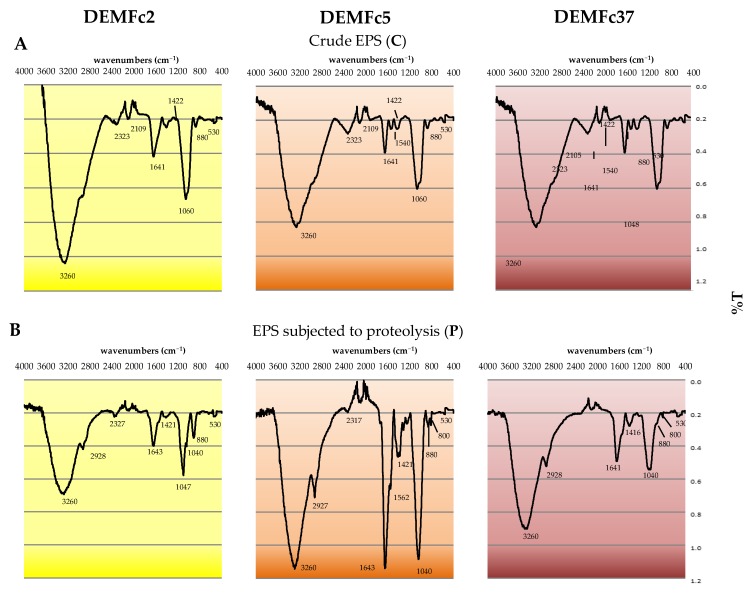
Infrared (FTIR) spectra of A. crude EPS (C) and B. proteolysed EPS (P) obtained from cultures of three *F. culmorum* strains: DEMFc2, DEMFc5, and DEMFc37.

The FTIR spectrophotometric analysis showed similarity but also big differences between the EPS of the analysed *F. culmorum* strains (Table 1, Figure 13). All of the EPS examined showed the presence of peaks, which confirms the presence of C=C ring carbons (1640 cm^−1^) and C-H aryl groups (860 cm^−1^) from the aromatic rings of phenol, aliphatic C-H groups (2928 cm^−1^), -C-O-, C-C, and –C-OH- groups (1040–1060 cm^−1^), and characteristic β-glycosidic bonds (880 cm^−1^) from sugar components. The presence of sugars in β-configurations was confirmed by FTIR analysis. Lack of signals at 930, 840 and 820 indicate the absence of sugars in α-configuration. 

#### 2.4.2. Molecular Weights of EPS Polysaccharidic Fractions, Glycosidic Linkages 

The EPS of the *F. culmorum* strains was substantially different in terms of the number of polysaccharidic subfractions: two for the EPS of the non-pathogenic DEMFc2 and DEMFc5 strains, and three for EPS of pathogenic DEMFc37 strain (Table 2). The molecular weight (MW) of the subfractions were determined as 1000–736 kDa and 16 kDa for DEMFc2 EPS; 74 kDa and 34 kDa for DEMFc5 EPS and 1000–736 kDa, 19-12 kDa, and 5kDa for DEMFc37 EPS. Thus, the EPS produced by the PGPR (DEMFc2) strain and by the pathogenic strain (Fc37) contain polysaccharides with a high molecular mass of about 1000-736 kD

Particularly large diversity of the MW of EPS subunits (from about 5 kDa to 2000 kDa) were recognized in *F. cocophillum* strain BCC2415 [16]. The molecular weight of the EPS polysaccharidic subfractions produced in the cultures of *F. culmorum* strains were much higher than the once determined for the *F. solani* DO7 EPS, which was merely 2.53 kDa. The polysaccharide of this *F. solani* strain most likely being homogeneous, as only one peak was observed in the chromatogram [6]. Mahapatra and Banerjee [44] described 190 kDa EPS of another *Fusarium* strain belonging to the *F. solani* species whose polysaccharide turned out to be a heteropolymer consisting of rhamnose and galactose. The type of linkages and percentage of glycosidic bonds in the polysaccharidic components of EPSs obtained from *F. culmorum* strains cultures was very diverse (Table 3).

The EPS obtained from the DEMFc5 culture exhibited a high diversity of type linkages, from terminal 6-d-Hex and Hex to 2-linkage Hex, 3-linkage Hex, 4-linkage Hex, 6-linkage Hex, and branched 3,6-linkage hexose residues. No branched hexose residues were observed only in the EPS of strain DEMFc2. Terminally-linked 6-deoxyhexose and mannose hexose I (Man) residues were detected, and most of these residues were found in the EPS of the DEMFc37 strain. 4-linkage Hex dominates in the EPS of DEMFc2, similar to the EPS of DEMFc5. Sugar monomers in fungal EPS are connected by different types of bonds, usually β(1→6) and β(1→3), but also α (1→3), α (1→2), and α (1→1) [87]. Polysaccharides with β-(1,3) and β-(1,4) linkage sugar monomers are considered to be particularly biologically active [35,36]. 

**Figure 14:**
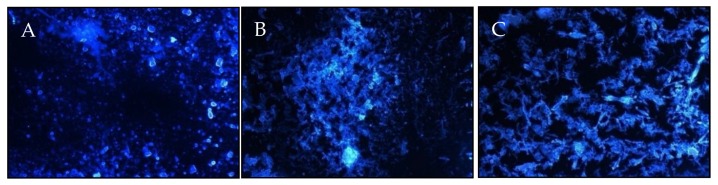
Visualization of polysaccharides containing β-glycosidic bonds (using Fluorescence Brightener 28 dye) in the ethanol precipitated EPS (EP) from cultures of *F. culmorum* strains: (**A**) DEMFc2, (**B**) DEMFc5, (**C**) DEMFc37.

The presence of β-glycosidic bonds in the ethanol precipitate of EPS (EP) obtained from the cultures of the three *F. culmorum* strains was visualized by staining with fluorescent dye Fluorescence Brightener 28 (Calcofluor White) dye (Figure 14). 

### 2.5. EPS Antioxidant Properties

**Figure 15:**
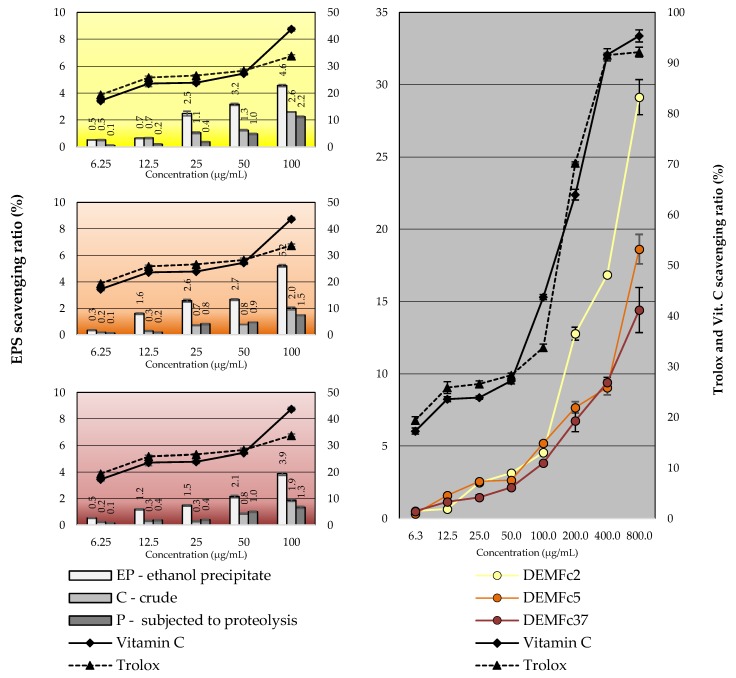
Scavenging ability (determined with the ABTS method) of the EPS obtained from cultures of three *F. culmorum* strains. (**A**). Comparison of ethanol precipitate EPS (EP), crude EPS (C), and proteolysed EPS (P) used in a concentration range of 6.25-100 µg/mL; (**B**). Ethanol precipitate EPS (EP) used in a concentration range of 6.25–100 µg/mL and in a range of 100–800 µg/mL. Standard deviations are shown as deviation bars (*n* = 9).

The three EPS preparations (EP, C, and P) had also antioxidant activity (Figure 15, Table 4). Low activity was detected at the EPS concentration range of 6.25–100 µg/mL, and the differences between the EPS activity from the strains was weakly visible (Figure 15A). In turn, ethanol precipitate EPS (EP) used in a concentration range of 100–800 µg/mL was characterized by strong antioxidant activity (Figure 15B).

Crude extract obtained from PDB culture of *F. oxysporum* isolated from *Otoba gracilipes* leaves was characterised by an over 50% of scavenging effect on DPPH [43]. All filtrates of cultures of 49 endophytic strains, including 18 from the *Fusarium* genus and 5 from the *Nectria* genus (*Fusarium* teleomorph), isolated from *Fritillaria unibracteata* displayed antioxidant activity, and significant positive correlations were found between total phenolic content (TPC) and antioxidant capacities (DPPH and ABTS) [89].

The EPS of the PGPR strain contained the greatest amounts of phenolic compounds, especially water-soluble, which is most likely associated with the highest antioxidant activity. It is well known that phenolic compounds are mainly responsible for antioxidant capacity of plant [12,90,91] and filamentous fungi [92], including Basidiomycota [93] and endophytic fungi [94,95]. Antioxidant properties of phenolic compounds are determined by their structure and may increase in the interaction with other phenolic compounds and in presence of polysaccharides, as demonstrated in studies of the antioxidant properties of a mixture of phenolic compounds with pectin [96].

The EPS of Fc2 was distinguished by particularly high antioxidant activity comparable to the antioxidant activity of EPS obtained from the Fc5 and Fc37 strain cultures (Table 4). The correlation coefficients clearly indicate the dependence of EPS antioxidant activity on the concentration of EPS in the solution. Similar correlation coefficient values were calculated for the standard antioxidant substances: vitamin C and Trolox. 

EPS are crucial bioactive metabolites of abundant endophytic *F. culmorum* strains and can be viewed as new antioxidant-rich resources. This *Fusarium* species requires further detailed research not only in terms of the properties and applications uses of its EPS but also other bioactive metabolites. It seems to have great but yet unexplored potential. The species *F. culmorum* is not mentioned in a comprehensive review [41] on the potential of endophytic fungi of the genus *Fusarium*.

Earlier studies of the three tested strains of *Fusarium culmorum* showed a clear diversity of their interaction with cereals, colonization of root [58,60] and root border cells [59] and the ability to synthesize toxins [58], CWDEs [63,64] and phytohormones [60,65]. The ability to induce plant resistance has been demonstrated for these *Fusarium culmorum* strains [58]. 

The first experiments carried out, in which the marker enzyme of plant resistance induction-phenylalanine lyase were determined in cereal plant, indicate a high elicitor potential of EPS obtained from the cultures of the tested strains of *F. culmorum*, and are being developed to demonstrate the role of EPS of these strains in the induction of plant resistance.

## 3. Materials and Methods 

### 3.1. Fungal Strains

The experiments were carried out on three *F. culmorum* (W.G. Smith) strains exerting distinct effects on the growth of rye, wheat, and barley [58]: one non-pathogenic growth promoting PGPF (plant growth promoting fungi) strain DEMFc2 (CBS 120098, NCBI DQ453700), one non-pathogenic strain detrimental to growth DRMO (deleterious rhizosphere microorganism) DEMFc5 (CBS 120101, DQ450880), and one pathogenic isolate DEMFc37. The DEMFc2 and DEMFc5 strains were isolated from the rhizosphere of healthy rye (*Secale cereale* L.), and the DEMFc37 (CBS 120103, DQ450878) strain was isolated from winter wheat (*Triticum aestivum* L.) [97]. Location: Poland, Lublin region, Latitude, longitude coordinates where collected 51.15 N; 22.34 E. Additional geographic data: Lublin Plateau, continental temperate climate (−20 °C to +30 °C). The strains were deposited in the Fungal Collection of the Department of Environmental Microbiology (DEM) and in the Centraalbureau voor Schimmelcultures Collections (CBS, Utrecht, The Netherlands). The nucleotide sequences (18S rRNA, internal transcribed spacer 1; 5.8S rRNA, internal transcribed spacer 2; and the 28S rRNA gene [a partial sequence]) of the isolates were deposited in the NCBI GenBank.

### 3.2. Optimization of Fungal Culture

Czapek-Dox medium [98,99] containing sucrose (30 g), peptone (7.5 g), dipotassium phosphate (1 g), magnesium sulphate heptahydrate (0.5 g), potassium chloride (0.5 g), and ferrous sulphate (0.01g) was used for cultivation of the fungal strains. The medium was sterilized by heating to 121 °C for 30 min. To find out the optimum incubation time, temperature, and medium pH for mycelial growth and EPS production, the fungi were grown at different time intervals (2–7 days), with different medium pH (4.5; 7.0; 9.5) and incubation temperature (12; 20; 28 °C). To investigate the requirement for additional nutrients for mycelial growth and EPS production, various carbon sources (sucrose, glucose, fructose, mannose) at different concentrations (3.75, 15, 30, 55, 187.5 g/L) and various nitrogen sources (peptone, yeast extract, ammonium sulphate, ammonium nitrate) at different concentrations (1.2, 2.5, 7.5, 30, 60 g/L) were used. The cultures were grown in an Innova chamber under controlled conditions of 8 hours of darkness and 16 h of lighting

### 3.3. Isolation of Exopolymeric Substances (EPS)

The culture was filtered through a cellulose filter and centrifuged at 11 000 rpm for 20 min. The supernatant was concentrated on a reverse osmosis column. The EPS was precipitated by addition of 96% ethanol:water (1:1 *v/v*) and left for 24 h at 4 °C. The 1:1 v/v ethanol:water ratio was selected in preliminary tests in which the efficiency of precipitation was checked using ethanol in a ratio of 1:1 to 1:4. Then, the precipitate was centrifuged at 11 000 rpm for 15 min, vacuum-dried in a lyophilizer (Freezone 6, Labconco, Kansas City, MO, USA) for 3 days, and stored in a desiccator until further investigation.

### 3.4. Deproteinization of EPS

The preparations were deproteinized using Proteinase K. 100 mg of the lyophilisate were weighed into a flask, and 30 mL of 50 mM phosphate buffer pH 7.5 and 0.5 mg of the enzyme were added. The incubation was carried out for 48 h at 37 °C. The obtained solutions were dialysed in a 12-kDa dialyser tube for 72 h at room temperature. Next, the solution was vacuum-dried in a lyophilizer for 3 days and stored in a desiccator until further investigation [100].

### 3.5. Determination of the Biochemical Composition in Water, Alkali, and Acid Solutions

#### 3.5.1. Obtaining Aqueous, Alkaline, And Acidic EPS Fractions by Step Fractionation

**Figure 16:**
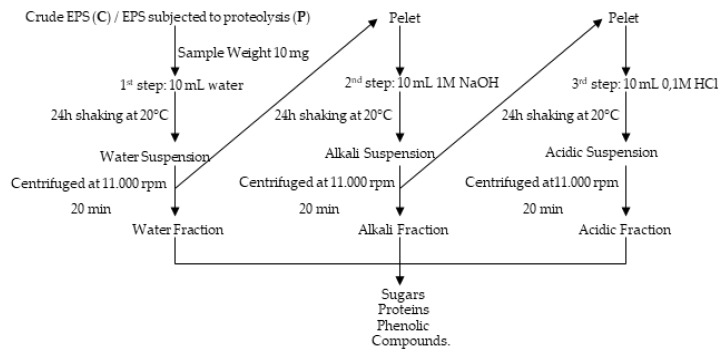
Scheme of fractionation of crude EPS (C) and EPS subjected to proteolysis (P) obtained from three *F. culmorum* strain cultures.

Aqueous, alkaline, and acidic fractions were obtained by three-step fractionation (Figure 16). The 1st step — the 10 mg of crude (C) or proteolysed (P) EPS obtained from three *F. culmorum* strain cultures: DEMFc2, DEMFc5, DEMFc37 were suspended in 10 mL of distilled water. After intensive shaking (120 rpm) for 24 hours at 20 °C, the water suspension was centrifuged at 11000 rpm for 20 min and the supernatant was subjected to EPS water fractions for determination of three components: proteins, phenolic compounds, and sugars.

The 2nd step: All water-insoluble pellets were suspended in 10 ml of 1M NaOH and shaken intensively (120 rpm) for 24 hours at 20 °C. The suspension was centrifuged at 11000 rpm for 20 min and the supernatant was subjected to the alkali EPS fractions. The 3rd step - All water and alkali-insoluble pellets were suspended in 10 ml of 0.1M HCl with intensive shaking (120 rpm) for 24 hours at 20 °C. The suspension was centrifuged at 11000 rpm for 20 min and the supernatant was subjected to the acidic EPS fractions.

#### 3.5.2. Preparation of Aqueous, Alkaline, and Acidic EPS Solutions by Direct Dissolution of EPS in Solvents

The aqueous, alkaline, and acidic EPS solutions were obtained by direct dissolution of EPS in each of the three solvents: water, 1M NaOH, and 0.1M HCl. The 10 mg of crude (C) or proteolysed (P) EPS were suspended in 10 mL of the above solvents. The suspensions were shaken (120 rpm) for 24 h at 20 °C. Next, they were centrifuged at 11000 rpm for 20 min and the supernatants were subjected to the aqueous, alkaline, and acidic EPS solutions. The content of soluble fractions of sugars, proteins, and phenolic compounds was determined in each solvent.

#### 3.5.3. Total Sugar

Total sugar was determined with the Dubois method. The 600 μL of 95% H2SO4 and 120 μL of 5% phenol were added to 200 μL of the sample. The mixture was heated in a boiling water bath for 5 min. After this time, the samples were cooled and the absorbance at 490 nm was measured against the control test [101]. 

#### 3.5.4. Protein

Proteins were determined using the Bradford method. Bradford working buffer (1 mL) was added to 100 μL of the sample and incubated for 2 min at room temperature. After this time, the absorbance at 595 nm was measured against the control test [102].

#### 3.5.5. Phenolic Compounds

Phenolic compounds were determined with the Folin-Ciocalteau method. The 250 μL of 50% (*v/v*) Folin-Ciocalteau reagent were added to 250 μL of the sample. After another 3 min, 500 μL of 7% Na_2_CO_3_ were added and incubated for 1 h. After this time, the absorbance at 680 nm was measured against the control test [103].

### 3.6. Visualization of Polysaccharides Using Confocal Microscopy

The 200 μL of Fluorescence Brightener 28 (F3543-1G, Sigma Aldrich, Steniheim, Germany) fluorescent dye was added to 1 mg of the EPS precipitates and incubated in the dark for 25 min. After this time, the dye solution was separated from the EPS by centrifugation, and then the pellet was applied to a slide and observed with excitation at 365 nm and emission at 435 nm under an Axivert 200M confocal microscope (Carl Zeiss Microscopy GmbH, Jena, Germany) equipped with a Zeiss LSM5 Pascal head 

### 3.7. Gas Chromatography

The preparations were hydrolysed in 2M TFA (trifluoroacetic acid) in water (100 °C, 4 h). The hydrolysates were dried in a desiccator and subjected to N-acetylation. The reagents were removed by evaporation under a stream of nitrogen. The preparations were reduced with NaBD_4_ (in H_2_O, room temperature, 18 h). After the reduction, the preparations were acidified (with glacial acetic acid) and dried. Boranes were removed by resuspension of the formulation in 5% acetic acid in methanol and evaporation in a stream of nitrogen and three-fold evaporation from methanol. The samples were peracetylated with acetic anhydride (Ac_2_O) and pyridine (100 μL, 1:1) at 100 °C for 30 min. The reagents were evaporated under a stream of nitrogen. The preparations were purified by extraction with water: chloroform (1:1, *v/v*). Peracetylated acetal (amino) acetates were recovered from the organic phase and dried by passing through a column of anhydrous sodium sulfate and drying under a stream of nitrogen. The preparations were analysed by gas chromatography coupled to a mass spectrometer (GC-MS), on a HP-5MS capillary column (30 m × 0.25 mm), using a 7890A gas chromatograph (Agilent Technologies, Santa Clara, CA, USA) and a 5975C XL EI/CI mass spectrometer with helium as a carrier gas (flow rate: 1 mL/min)( Agilent Technologies, Santa Clara, CA, USA). Temperature program: 150 °C for 5 min built up to 310 °C at 5 °C min^−1^; the end temperature was maintained for 10 min [100].

### 3.8. Determination of the Size of Polymers in Preparations after Proteinase K Treatment/Molecular Weights of Polysaccharidic Subfractions of EPSs

The average molecular mass of the EPS was determined by gel permeation chromatography (GPC) using a Sepharose 6BCL matrix (CL6B200, Sigma Aldrich, Steinheim, Germany). The sample was dissolved in 1 M NaOH (5 mg/mL). The material (0.5 mL) was eluted from the column (80 cm × 0.7 cm) with a 1 M NaOH solution (fractions of 40 drops), at a flow rate of 0.25 mL/min. [36,100].

### 3.9. FTIR Spectroscopy Analysis 

FTIR analysis was carried out on the EPS lyophilisates. FTIR spectra were measured in the frequency range of 4000–500 cm^−1^ with the resolution of 4.0 cm^−1^ and a scanning rate of 320 scans/s. The FTIR spectrum of the EPSs were measured on a Cary 630 spectrometer (Agilent, Santa Clara, CA, USA).

### 3.10. Determination of the Presence of Glycosidic Bonds

The linkage position in the polysaccharides was established by methylation according to the method proposed by Hakomori and extraction of permethylated products into chloroform, followed by acid hydrolysis (2M TFA, 100 °C, 4 h), reduction with NaBD_4_, and per-acetylation. The partly methylated alditol acetates obtained were analysed by GC–MS [36,88]. 

### 3.11. Antioxidant Properties-ABTS Method

The ABTS stock solution was prepared by dissolving 76 mg ABTS in 20 mL 0.1M PBS buffer. Then, 13.2 mg of K_2_S_2_O_8_ were added to oxidize ABTS. The solution prepared in this way was incubated in the dark for 16 h. After incubation, 10 μL of the sample was added to 990 μL of working ABTS solution and the mixture was incubated at room temperature for 5 minutes. Absorbance was read at a 734 nm wavelength. The free radical scavenging effect was calculated using the following Equation (1):

***% scavenging of free radicals ABTS•+ = ((A0 –A1)/A0) x100*** (1)

where A0-absorbance of the control sample and A1-absorbance of the tested sample. Vitamin C and Trolox were used as standards at concentrations of 6.25; 12.5; 25; 50; 100, 200, 400, 800 µg/mL for every measurement [104,105].

### 3.12. Statistical Analysis

The experiments were performed three times with three samples per each treatment and the data were expressed as means ± SD (shown as deviation bars) calculated from these experiments. Standard deviations were determined using Microsoft Excel 2016 (Microsoft Corp., Redmond, WA, USA). The Pearson correlation coefficient (*R*) and the linear regression coefficient (*R*2) were determined (using Microsoft Excel 2016). The data were subjected to the one-way analysis of variance (ANOVA) followed by a Tukey’s post hoc test, with the significance evaluated at *p* < 0.05 [106]. The principal component analysis (PCA) was performed in the MVSP3.21 package. The analyses of PCA were based on the average values of three independent replicates.

## 4. Conclusions and Future Perspective

For the first time, the EPS of fungi belonging to the cosmopolitan endophytic species *Fusarium culmorum* was obtained and characterized. In addition, the similarities and differences in EPS production and properties between strains of this species exerting different effects on cereals (growth-promoting, harmful, and pathogenic) were determined.

Clear differences were observed in the dynamics of EPS production, optimal conditions for this process, and the composition and antioxidant properties among EPS produced by the growth-promoting non-pathogenic *F. culmorum* strain and EPS obtained in cultures of the harmful and pathogenic strains. The pathogen synthesized EPS at much higher concentrations than those produced by the non-pathogenic strains. The EPS of the pathogen contains the highest amounts of sugars, with mannose as the dominant sugar in the polysaccharide portion of EPS. Three subfractions were found only in the pathogen EPS and two substractions were determined in the EPS of the non-pathogenic strains. The lightest 5 kDa subfraction was only detected in the pathogen EPS. In contrast, the EPS of the PGPR strain contained the greatest amounts of phenolic compounds, especially water-soluble. The EPS of this non-pathogenic strain had significantly greater antioxidant activity than the EPS of the other strains, which is most likely associated with the highest content of phenolic compounds in this EPS.

These differences indicate that EPS may be a factor responsible for the intensity of colonization and a determinant of the course of interactions of individual *Fusarium* strains with the plant. The pilot study indicates that the EPS of these strains may act as metal chelating compounds and inducers of plant resistance; therefore, they can play a key ecological role not only in shaping the microbiome of the soil environment and its interaction with the plant, but also in medical (as antioxidants), agricultural (as biofertilizers and biocontrol factors) applications as well as in environmental protection (in bioremediation of metal-contaminated environments). 

## Figures and Tables

**Table 1:** Distribution of the main peaks at FT-IR spectra of proteolysed EPS (**P**) obtained from cultures of three *F. culmorum* strains: DEMFc2, DEMFc5, and DEMFc37.

Wavenumbers [cm^−1^]	Functional Groups Identified
3260	-OH; stretching vibrations of OH groups related to inter-residue hydrogen linkages
2928	C-H; stretching vibrations of aromatic and aliphatic C-H groups
2320	N-H groups; stretching vibrations of N-H groups from protein
1643	C=C ring carbons; stretching vibrations from the hexameric ring carbons (phenolic compounds or amino acids)
1420	-OH groups; stretching vibrations from alcohol -OH groups
1040–1060	-C-O- and -C-C- stretching vibrations and –C-OH- bending vibrations
880	the β-glycosidic linkages from sugars
860	C-H aryl; bending vibrations from aromatic rings

**Table 2:** Molecular weight distributions of proteolysed EPS (P) obtained from cultures of three *F. culmorum* strains.

Peak No.	DEMFc2	DEMFc5	DEMFc37
	Mass (kDa)	Mass (kDa)	Mass (kDa)
1	1000–736	74	1000–736
2	16	34	19–12
3	-	-	5

**Table 3:** Percentage of glycosidic bonds in polysaccharidic components of EPSs (P) from *F. culmorum* strains (methylation analysis according to the Hakomori procedure [88].

Ret. Time	Glycosidic Linkage	DEMFc2	DEMFc5	DEMFc37
11.14	(terminal) 6-d-Hex(1→	-	3,4	-
12.51	(terminal) Hex(1→(Man)	21,5	17,5	50,4
14.40	→2)Hex(1→	Traces	16,8	24,5
14.45	→3)Hex I(1→	25,3	10,3	-
14.54	→4)Hex(1→	53,2	29,0	5,5
14.88	→3)Hex II(1→	-	3,1	7,2
15.10	→6)Hex(1→	-	4,8	-
17.03	→3,6)Hex(1→	-	15,2	12,3

**Table 4:** Scavenging ability (determined with the ABTS method) of the EPS obtained from cultures of three *F. culmorum* strains. comparison of ethanol precipitate EPS (**EP**), crude EPS (**C**), and proteolysed EPS (**P**) used in a concentration range of 6.25–100 µg/mL and ethanol precipitate EPS (**EP**) used in a concentration range of 6.25–100 µg/mL and in a range of 100–800 µg/mL. Data are presented as means ± Standard deviation (*n* = 9).

	Scavenging Ratio (%)										
Concentration (µg/mL)	DEMFc2			DEMFc5			DEMFc37			Vit. C	Trolox
	EP	C	P	EP	C	P	EP	C	P		
**6.25**	0.52 ± 0.02	0.50 ± 0.04	0.10 ± 0.02	0.33 ± 0.03	0.20 ± 0.02	0.10 ± 0.03	0.52 ± 0.01	0.19 ± 0.02	0.10 ± 0.02	20.12 ± 0.68	19.13 ± 0.61
**12.5**	0.63 ± 0.03	0.66 ± 0.04	0.20 ± 0.03	1.59 ± 0.06	0.29 ± 0.03	0.20 ± 0.01	1.15 ± 0.04	0.29 ± 0.03	0.36 ± 0.04	24.30 ± 1.16	25.32 ± 0.49
**25**	2.50 ± 0.17	1.00 ± 0.06	0.36 ± 0.03	2.55 ± 0.11	0.69 ± 0.03	0.85 ± 0.05	1.40 ± 0.05	0.30 ± 0.03	0.40 ± 0.04	25.77 ± 0.64	26.40 ± 0.32
**50**	2.25 ± 0.08	1.20 ± 0.05	0.95 ± 0.04	2.58 ± 0.06	0.79 ± 0.04	0.96 ± 0.02	2.03 ± 0.09	0.85 ± 0.04	0.98 ± 0.05	27.40 ± 0.41	27.70 ± 0.65
**100**	2.94 ± 0.09	2.60 ± 0.02	2.20 ± 0.06	3.08 ± 0.02	1.90 ± 0.06	1.50 ± 0.02	2.99 ± 0.11	1.85 ± 0.09	1.36 ± 0.07	42.8 ± 0.67	33.90 ± 0.51
**200**	12.77 ± 0.75	IS	IS	7.68 ± 0.39	IS	IS	6.75 ± 0.44	IS	IS	63.98 ± 0.20	70.23 ± 1.05
**400**	16.85 ± 0.35	IS	IS	9.06 ± 0.52	IS	IS	9.41 ± 0.15	IS	IS	91.62 ± 0.39	91.55 ± 1.20
**800**	29.14 ± 1.55	IS	IS	18.62 ± 1.02	IS	IS	14.41 ± 1.21	IS	IS	95.35 ± 0.97	92.14 ± 1.19
**R^2^**	**+0.629**	**+0.791**	**+0.654**	**+0.664**	**+0.75**	**+0.886**	**+0.721**	**+0.676**	**+0.851**	**+0.874**	**+0.840**

## Abbreviations

EPS	exopolymeric substances
DEM	department of environmental microbiology
PGPF	plant growth-promoting fungi
DRMO	deleterious rhizosphere microorganisms
kDa	kilodaltons
FTIR	infrared spectroscopy
PS	polymeric substances
UV	ultraviolet
DMSO	dimethyl sulfoxide
CWDE	cell wall degrading enzymes
PCA	principal component analysis
FTGM	fungal-types galactomannansfungal-type
Hex	hexose
Man	mannose
TPC	total phenolic content
DPPH	2,2-diphenyl-1-picrylhydrazyl
ABTS	2,2′-azino-bis (3-ethylbenzothiazoline-6-sulfonic acid)
Vit. C	vitamin C
